# Should I Stay, or Should I Go? Job satisfaction as a moderating factor between outcome expectations and entrepreneurial intention among academics

**DOI:** 10.1007/s11365-021-00744-8

**Published:** 2021-06-21

**Authors:** Richard Blaese, Schneider Noemi, Liebig Brigitte

**Affiliations:** 1 Department of Social Psychology, University of Basel, Missionsstrasse 64A, CH-4055 Basel, Switzerland; 2 University of Applied,Sciences Northwestern Switzerland, Olten, Switzerland

**Keywords:** University spin-offs, Entrepreneurial intention, Academic entrepreneurship, Perceived utility, Job satisfaction, Social cognitive career theory, L26, M13, O32

## Abstract

Both psychological and entrepreneurship research have highlighted the pivotal role of job satisfaction in the process of entrepreneurial career decisions. In support of this, mounting evidence point to inter-relationships between entrepreneurial intention, job satisfaction, and organizational commitment. Prior research operationalized entrepreneurial careers as an escape from poor work environments; thus, there is a lack of understanding regarding how job-satisfaction can trigger entrepreneurship within and related to the environment of universities. This study, draws on Social Cognitive Career Theory and the concept of entrepreneurial intention, to address whether the role of job satisfaction is a moderating factor between outcome expectations and entrepreneurial intention. Furthermore, we examine to what extent (I) entrepreneurial intention and (II) spin-off intention are determined by certain outcome expectations and perceived behavioral control. To address these questions this study examined academic researchers in specialized and non-technical fields and builds on a survey of 593 academic researchers at Swiss Universities of Applied Science. The results showed that outcome expectations are a significant predictor for entrepreneurial intentions, in general, and spin-off intentions, in particular. A multi-group analysis corroborated that job satisfaction operates as a motivational factor in entrepreneurial transition and interactions with entrepreneurial outcome expectations. In conclusion, the concept of job satisfaction and Social Cognitive Career Theory were powerful constructs to better the understand the process of entrepreneurial career decisions by academic researchers.

## Introduction

Academic entrepreneurship is widely recognized for its contribution to economic, regional, and innovation development (Audretsch, 2014; Block et al., 2017; Fini et al., 2018; Guerrero et al., 2015; Shane, 2004; Stuetzer et al., 2018). As a specific form of entrepreneurship,^1^ academic entrepreneurship refers to the “commercial application of academic research” (Abreu & Grinevich, 2017, p. 764). According to the right of universities to claim the ownership of intellectual property stemming from research (e.g., caused by the US Bayh-Dole Act of 1980), the notion of the ‘entrepreneurial university’ emerged in the late 1990s (Etzkowitz & Leydesdorff, 1998). The entrepreneurial university is comprised of services and tasks that go beyond research, teaching, and redefines the organizational outlook of departments as well as the interaction between research, education and innovation.

Spin-off activities are recognized as a central element of the so-called ‘third mission’ of universities (Etzkowitz, 2003). Universities seek to develop policies and instruments that encourage entrepreneurial careers of their academic researchers and support spin-offs to commercialize research as a specific form of academic entrepreneurship. Within the entrepreneurial literature, there is ample evidence that universities are key actors in shaping and influencing favorable entrepreneurial ecosystems, for example by creating an entrepreneurial culture, inaugurating technology transfer offices and providing infrastructure and incentives for entrepreneurial activities (Etzkowitz, 2003, 2014, 2017; Huyghe & Knockaert, 2015; Kirby et al., 2011; Meek & Wood, 2016; Miller et al., 2018). Although the literature on academic entrepreneurship has focused primarily on exogenous factors such as socio-organizational conditions affecting entrepreneurial decision-making (Feola et al., 2017; Huyghe & Knockaert, 2015; Kirby et al., 2011; Miranda et al., 2017), empirical research identifying endogenous, motivational factors such as job-satisfaction for entrepreneurial careers of academics are remain limited.

Academic researchers traditionally select among the following career options: (a) remaining in research positions, (b) moving to industry or services, or (c) become a full or part-time entrepreneur (Murray, 2004). Crucial to the entrepreneurial process is the deliberate initiation of entrepreneurial activities that lead to, in the case of academic entrepreneurship, the creation of spin-off companies. It is generally acknowledged that entrepreneurship represents an intended career decision based on motivational factors such as desirable outcome expectations and perceived behavioral control—the individual’s perception of whether an entrepreneurial action would be feasible (Douglas & Shepherd, 2002; Tran & Korflesch, 2016; Shane, 2004). By using outcome expectations and an agent perspective, Social Cognitive Career Theory (SCCT) (Lent et al., 2002, 1994) is a valuable construct previously used in research to shed light on the contributors of entrepreneurial motivation (Kassean et al., 2015; Liguori et al., 2018; Segal et al., 2002; Tran & Korflesch, 2016).

The concept of entrepreneurial intention is widely used to investigate the earlier stages of academic entrepreneurship (Goethner et al., 2012; Hossinger et al., 2020; Miranda et al., 2018; Obschonka et al., 2015). Entrepreneurial decision-making is understood as a form of career choice, made in a unique organizational context, based on individual, socio-cognitive, and environmental characteristics (D’este & Perkmann, 2011; Lam & Campos, 2015; Lam, 2015; Rizzo, 2015). Limited studies have explored the motivating factors driving academics to engage in entrepreneurship (e.g., Abreu & Grinevich, 2013; Guerrero & Urbano, 2014). What research has been conducted suggests a strong interconnection between propensity to participate in entrepreneurship activities and job satisfaction – the expression of the fit between job expectations and current employment conditions, organizational commitment and entrepreneurial decision-making (Singh & Onahring, 2019).

,Previous research demonstrates that through a low level of commitment, entrepreneurial behavior becomes more attractive if employment conditions are perceived as dissatisfying (Guerra & Patuelli, 2016; van Dick et al., 2004; Werner et al., 2014). As a so-called push-factor, job-dissatisfaction accelerates the transmission from wage employment to self-employment. For example, Guerra and Patuelli (2016) reported that pecuniary and nonpecuniary job satisfaction significantly affects transition to self-employment and job changes in Switzerland. This study also showed that the transition probability was positively affected by the level of education, suggesting a higher likelihood of well-educated individuals leaving unsatisfying employment.

Academic spin-offs, which are linked to the commercial knowledge transfer of universities, are usually considered from a scientist's perspective as the continuation of their academic career, rather than a career exit (Shane, 2004; Lam, 2015). Therefore, in contrast to the research discussed above, evidence also exists that high job satisfaction has a positive effect on taking ownership of the translation of the organization's values and goals (Neessen et al., 2019; Tang et al., 2019). In the context of the entrepreneurial university, spin-off activities are emphasized as organizational goals—and thus are hypothesized to be an expression of the individual's organizational commitment. Despite this, entrepreneurship literature is still limited with respect to systematic research investigating the interaction between job satisfaction and entrepreneurial career goals in terms of spin-off activities among academic researchers. Since entrepreneurship in terms of knowledge transfer embodies unique outcome expectations (e.g., personal and financial gain or career enrichment), which contrasts with extramural forms of entrepreneurship (Lam, 2015; Hossinger et al., 2020; Shane, 2004), additional research is warranted to evaluate whether job satisfaction acts as a moderator of entrepreneurial intention, spin-off intention, or both.

By focusing on spin-off outcomes, academic entrepreneurship research thus far has largely overlooked the fact that there are various forms of entrepreneurial activities among academics, and not all are necessarily geared towards knowledge transfer. This issue is also reflected in existing research with some studies evaluating academic entrepreneurship in the form of spin-off intention to commercialize research (Brettel et al., 2013; Goethner et al., 2009, 2012; Huyghe & Knockaert, 2015; Obschonka et al., 2012, 2015). Other studies have taken a broader perspective and simply examined entrepreneurial intention (Foo et al., 2016; Moog et al., 2015), or framed it as a way to move from wage employment to self-employment outside the organization. Taken together, we argue that the multitude ways that entrepreneurship has been operationalizing among academics makes it difficult to compare study results and generate generalizations in the field.

There is little research examining the role of socio-environmental conditions, such as job satisfaction and job dissatisfaction, as a motivational driver of different forms of entrepreneurial modes among academics (Singh & Onahring, 2019). In this study, we seek to address this knowledge gap by evaluating the relationship between job satisfaction and entrepreneurial intention as well as between job satisfaction and spin-off intention. Specifically, we investigate whether spin-off activities are more attractive to academics when job satisfaction is high, or if low job satisfaction drives rates of pursuing entrepreneurship in terms of an academic career exit strategy. Building on Social Cognitive Career Theory, which is a valuable framework for assessing the influence of organizational variables on (entrepreneurial) career decisions (Kassean et al., 2015; Liguori et al., 2018; Segal et al., 2002; Tran & Korflesch, 2016), this study sheds empirical light on entrepreneurial career-path of academics by combining the above research questions. The motivational mechanisms behind entrepreneurial decisions were investigated according to a survey of Swiss academics from various disciplines at the Swiss Universities of Applied Science (UAS). With a sample of 593 participants, we test the proposed research questions using structural equation modeling.

This study contributes original data to the emerging research on academic entrepreneurship. First, it addresses the motivational role of job (dis-)satisfaction in the entrepreneurial process for academics. Second, it highlights the role of outcome expectations and perceived behavioral control in modulating the entrepreneurial career decisions of academics, thus offering more in-depth insight into the interplay between job satisfaction and the scientists' outcome expectations and commitment to entrepreneurship. Third, it develops an empirical application of SCCT in academic entrepreneurship research by demonstrating empirical differences between academic entrepreneurship and entrepreneurial academics in terms of outcome expectations. Overall, this study contributes to a greater understanding the dynamics driving academics to participate in entrepreneurial activities.

## Theoretical framework

### Motivational drivers of entrepreneurship in academia

Entrepreneurial motivations are considered to be the initial inspiration for an individual to launch a new business (Shane et al., 2003). Prior research explicitly addressed motivational factors for spin-off activities (Antonioli et al., 2016; Guerrero & Urbano, 2014; Lam, 2015; Houweling & Wolff, 2019; Morales-Gualdrón et al., 2009; Shane et al., 2003) and posit that socio-organizational factors have a significant influence on the motivation of academics to become entrepreneurial (Feola et al., 2017; Miranda et al., 2017; Morales-Gualdrón et al., 2009). For example, in their empirical study of Spanish academic entrepreneurs, Morales-Gualdrón et al. (2009), identified the following factors to be major drivers of entrepreneurial motivation: personal, entrepreneurial potential (i.e., entrepreneurial opportunity), scientific knowledge, and availability of resources, incubator organization and social environment (i.e., social norms and attitudes). In an in-depth interview-based study, Guerrero and Urbano (2014) used nascent academic entrepreneurs to analyze individual motivations. Their findings showed that academic entrepreneurs define various outcomes, including technology diffusion, technology development, financial gain, public service, and peer motivation.

Lam (2015) offers a conceptual framework for the motivation of researchers to participate in spin-off activities, which included of three types of motivation: ‘Gold’ (as for financial rewards); ‘Ribbon’ (as a reward for reputation/career); and ‘Puzzle’ (as intrinsic satisfaction). Lam (2015) also stresses that the majority of academic entrepreneurs participating in spin-off creations are motivated by intrinsic and reputation-related factors rather than pecuniary expectations. The synthesis of the literature mentioned above would suggest that the outcome expectations associated with spin-off activities are mainly related to improving current employment opportunities in academia.

Individual personality traits such as self-efficacy (Chang & Edwards, 2015; Huyghe & Knockaert, 2015; Zhao et al., 2005), attitudes (Goethner et al., 2012; Miranda et al., 2017), entrepreneurial passion (Obschonka et al., 2015) and job dissatisfaction have been repeated identified in the literature as key drivers of entrepreneurship (Brockhaus, 1982; Chang & Edwards, 2015; Guerra & Patuelli, 2016; Jeong & Choi, 2017; Singh & Onahring, 2019). In their review, Singh and Onahring, p. 2 (2019) defined job satisfaction as “the difference between the quantum of rewards received by employees and the amount they believe they should receive”. Based on previous research, the authors postulated a research model that assumed a positive relationship between job satisfaction, organizational commitment and entrepreneurial intention. Although job dissatisfaction can act as a push factor for entrepreneurial intentions (Brockhaus, 1982; Guerra & Patuelli, 2016), job satisfaction can alternatively strengthen individual's proactivity, intrapreneurship (Neessen et al., 2019) and organizational commitment (Tang et al., 2019), such as the implementation of an entrepreneurial mission.

### The Social Cognitive Career Theory (SCCT)

When studying entrepreneurial career decisions, scholars have widely acknowledged entrepreneurial intention to be the first step in a long entrepreneurial process and to be the ‘best’ predictor of entrepreneurial behavior (Bird, 1988; Krueger et al., 2000). Entrepreneurial intention reflects a mental process that accompanies the planning and implementation of entrepreneurial actions (Boy & Vozikis, 1994; Tran & Korflesch, 2016). To date, researchers have applied several theoretical models to study the formation of entrepreneurship intention. These include the Model of Entrepreneurial Events (SEE) (Shapero & Sokol, 1982), the Theory of Planned Behavior (TPB) (Ajzen, 1991, 2011; Tornikoski & Maalaoui, 2019), the Social Cognitive Career Theory (SCCT) that analyzes career choices (Lent et al., 1994, 2002). SCCT considers environmental (see Liguori et al., 2018; Tran & Korflesch, 2016) and motivational influences, such as outcome expectations and feasibility beliefs in form of self-efficacy to predict career decisions. In comparison to other theoretical approaches, the SCCT is considered to have a number of advantages. For example, SCCT defines precise intention predictors that are not as abstract as represented by other intentional models (e.g., perceived desirability in SEE versus outcome expectations in SCCT) (Tran & Korflesch, 2016). SCCT postulates that career goals are determined by the assessment of cognitive-individual factors (e.g., self-efficacy, ‘I will be able to do this’) and associated outcome expectations (‘If I do this, then what will be the outcome?’) (Lent et al., 1994, p. 83). By evaluating past behavior, individuals gain an understanding of social environmental factors, their cognitive capabilities (e.g., domain-specific self-efficacy) to shape future career goals. Scholar frequently employed SCCT as a theoretical framework to help explain career choices (Lent et al., 2008) based on individual cognitive factors (Lent et al., 2002) originating from Bandura's general socio-cognitive theory (1986). SCCT has been empirically applied in a variety of contexts (Chang & Edwards, 2015; Lent et al., 2002, 2008; Rogers & Creed, 2011), leading Liguori et al. (2018) to recommend it as a valid theoretical framework for investigating entrepreneurial career goals.

### Development of hypotheses

In line with SCCT, the present contribution focuses on entrepreneurial and spin-off intention as a career choice, by assuming outcome expectations and self-efficacy beliefs to influence entrepreneurial decision making. Both self-efficacy and perceived behavioral control refers to the individual’s perception of whether or not an action would be difficult to perform (Ajzen, 2002). In their literature review, Tran and Korflesch (2016) argue that the construct of self-efficacy in SCCT was conceptually similar to the constructs of perceived behavioral control in TPB (Ajzen, 1991) and perceived feasibility in SEE, “as they are all about perception of capability to start a social venture” (Tran & Korflesch, 2016, p. 23). According to Bandura, self-efficacy refers to the individual’s “judgment of their capabilities to organize and execute courses of actions required to attain designated types of performance” (Bandura, 1986, p. 391) and thus to one’s own perceived abilities. In the framework of SCCT, self-efficacy beliefs are posited to predict career goals, and influences outcome expectations, as people expect outcomes that are more desirable in activities where they consider themselves effective (Bandura, 1986). Ajzen (2002) considered Bandura's (1986) concept of self-efficacy (dealing with ease or difficulties in task performance) as part of a superordinate construct of perceived behavioral control. In the field of academic entrepreneurship, studies show that both perceived behavioral control and self-efficacy are strong predictors of entrepreneurial intentions (Boy & Vozikis, 1994; Goethner et al., 2012; Guerrero et al., 2008; Huyghe & Knockaert, 2015; Obschonka et al., 2012, 2015).

Informed by prior research, we believe that perceived behavioral control will be positively associated with entrepreneurial intention. The following hypotheses are made according to SCCT and the larger body of literature:

(H1a) Among academics, perceived behavioral control positively influence entrepreneurial intention.

(H1b) Among academics, perceived behavioral control positively influence spin-off intention.

Outcome expectations are personal beliefs about possible and imaginary consequences of specific behaviors, which are considered to be fulfilled as a result of a specific action (Lent et al., 1994). SCCT, therefore, assumes that individuals are more willing to act if they believe that the associated outcome expectations are more achievable (Liguori et al., 2018). Based on SCCT and the expectation theory of Vroom (1964), outcome expectations are a key factor to predict career goals. Expectation theory states that individuals are motivated to participate in an activity if they believe that the goal is worth the effort and that there is a way to realize the goal. In terms of entrepreneurship, outcome expectations result from a global assessment of expected efforts and the resulting benefits (Douglas & Shepherd, 2000). According to literature, one will favour an entrepreneurial career if the expected profits from entrepreneurship are higher than the sum of the expected future benefits from employment (Goethner et al., 2012, p. 630). As outlined above, the literature considers various motivations that may encourage academics to become entrepreneurial (Morales-Gualdrón et al., 2009; Lam, 2015; Guerrero & Urbano, 2014). In their review of literature, Hossinger et al. (2020) suggested that academics choose entrepreneurial activities in order to pursue an intrinsic source of rewards, such as independence, sense of achievement, inner saturation and self-realization or external rewards, and academic benefits from the creation of spin-off companies. Academics may consider spin-off activities as an opportunity to obtain academic reputation (Lam, 2015) or to gather resources, such as access to financial funding or new infrastructure to support research (Hossinger et al., 2020).

We expect that outcome expectations, in terms of pecuniary gains, satisfaction, autonomy, and quality of life, are predictors of both entrepreneurial intentions and spin-off activities. Previous research on entrepreneurship has shown that certain expectations (e.g., pecuniary and non-pecuniary satisfaction) predicted entrepreneurial decisions (Guerrero & Urbano, 2014; Miranda et al., 2017). For example, expected reputation and financial gains indirectly influenced spin-off intentions of academic researcher (Goethner et al., 2012; Miranda et al., 2017; Lam, 2015). Thus, we pose the following hypotheses:

(H2a) Among academics, outcome expectations positively influence entrepreneurial intention.

(H2b) Among academics, outcome expectations positively influence spin-off intention.

### Job satisfaction as a two-way moderator

In 2019, Singh and Onahring (2019) reviewed various theoretical frameworks depicted the interrelationships between job satisfaction, organizational commitment, and entrepreneurial intention. For example, Vroom (1964) defined job satisfaction as an affective orientation of the individual towards his current employment conditions. Based on Singh and Onahring's (2019) assumption, job satisfaction is an indicator and measure of the fulfillment of work-related expectations and personal needs. The literature notes that job satisfaction could be affected by various organizational conditions, such as perceived autonomy, job content, job flexibility, social benefits, career prospects, and interpersonal relationships (Agho et al., 1993; Shvets, 2018). Although employees who are satisfied with the conditions tend to be more committed towards their organizational norms (Tang et al., 2019), research has demonstrated that job dissatisfaction positively affects career decisions that include increased entrepreneurial activities (Chang & Edwards, 2015; Guerra & Patuelli, 2016; van Dick et al., 2004; Werner et al., 2014). It has been argued that, under certain circumstances, the transition to entrepreneurship is a kind of exit strategy from current employment (Brockhaus, 1982). For example, those employees who are dissatisfied with their employment conditions would expect improvements from a change to self-employment, accompanied by greater economic benefits than those remaining in their current employment (Guerra & Patuelli, 2016). In general, job satisfaction is an indicator and a driver for the evolution of new outcome expectations and career goals, as it creates a subjective framework for both interpretation and behavior.

**Fig. 1:**
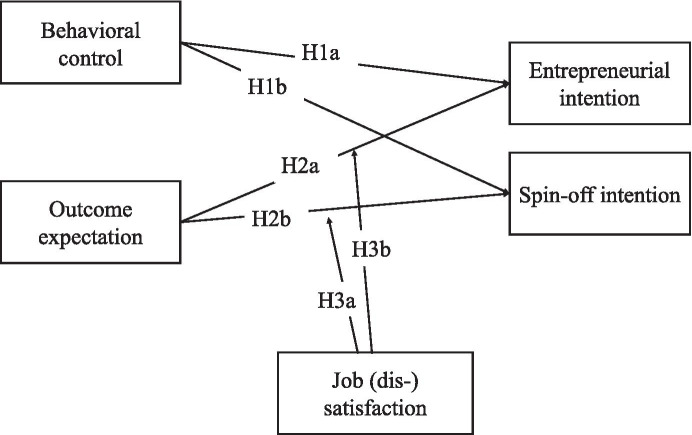
Conceptual research model. Hypothesized predictors of an entrepreneurial intention and spin-off intention and hypothesized moderating effects of entrepreneurial job (dis-)satisfaction on intentions within the SCCT- framework

SCCT states that career goals are affected by personal, environmental, and situational factors (Tran & Korflesch, 2016), including both objective and subjective environmental conditions such as job satisfaction. Subjective environmental conditions influence the individuals' interpretation regarding opportunities, resources, barriers, and pecuniary benefits (Lent et al., 1994). According to prior research, satisfied employees display higher levels of organizational commitment, higher productivity, and more punctual as well as efficient behavior (Lumley et al., 2011; Tang et al., 2019). A higher level of organizational commitment is related to the desire to pursue a career within the organization (Feinstein & Vondrasek, 2001; Meyer et al., 2002). We argue that entrepreneurial academics with a high level of job satisfaction show a greater propensity to commercialize their research by developing spin-offs that align with the aims of Entrepreneurial Universities (Etzkowitz, 2017). Based on a psychological contract (Rousseau, 1995), employees try to implement the organization’s goals in a meaningful way and align their behavior accordingly. For example, Huyghe and Knockaert (2015) demonstrated that the entrepreneurial mission of the university has a positive effect on spin-off intentions. Obschonka et al. (2012) showed that academics who feel attached to their university are more likely to follow institutional norms in terms of entrepreneurial goals. As entrepreneurial universities create a specific environment to encourage spin-off activities and practices that promote the commercialization of R&D (Etzkowitz, 2017; Kirby et al., 2011), entrepreneurial academics will feel committed to them in order to gain reputation within their organization (Lam & Campos, 2015). Thus, researchers with high job satisfaction will automatically weigh their entrepreneurial outcome expectations more positively than academics with low job satisfaction to pursue an entrepreneurial career within and promoted by the ecosystem of entrepreneurial universities. This study assumes in particular that a high level of job satisfaction has a moderating effect on the relationship between outcome expectations and spin-off intention of academics. Accordingly, the following hypothesis can be developed (Fig. 1):

(H3a): Job satisfaction has a moderating effect on the relationship between outcome expectations and spin-off intention, such that when job satisfaction is high the relationship is stronger and when job satisfaction is low the relationship is lower.

In contrast to the effects of job satisfaction, scholars widely acknowledged that high job dissatisfaction – i.e., the experience of frustration over unfulfilled expectations, increases the rate of individuals leaving their job (van Dick et al., 2004; Werner et al., 2014; Werner & Moog, 2007) and decreases their degree of organizational commitment (Singh & Onahring, 2019). The study of job dissatisfaction is now core to entrepreneurship research, as the creation of businesses such as start-ups is perceived as a way to escape poor working conditions and thus as an alternative to the current employment (Brockhaus, 1982; Lee et al., 2011; Singh & Onahring, 2019). As associated with the construct of outcome expectations, individuals compare costs and benefits when choosing (an entrepreneurial) goals (Lent et al., 2002). Therefore, job dissatisfaction as an indicator that describes the individual's perception of the perceived disadvantages of remaining in the current job, is triggered by a low level of autonomy, financial resources or pay, lack of career options, poor opportunities for advancement within the organization, and excessive workload. Morales-Gualdrón et al. (2009) noted that there are numerous motivators at the organizational level for academic researchers to leave their university positions to start a new business; these factors include dissatisfaction regarding current workloads, high bureaucracy, and low-risk orientation of the parent organization. Guerrero and Urbano (2014) observed that ‘motivating peers’ either came from outside the university or were perceived as rebels within the university, suggesting that universities have either so far not sufficiently supported academic entrepreneurship or that these individuals do not feel committed to their organizations and thus encouraged to commercialize their research.

Given empirical evidence for a positive relationship between dissatisfaction with current employment and individuals' intention to pursue an entrepreneurial career (Werner et al., 2014; Guerra & Patuelli, 2016), we argue that job dissatisfaction moderates the relationship between outcome expectations and entrepreneurial intention. Accordingly, we expect that entrepreneurial academics who are encouraged by the desire to leave university due to job dissatisfaction will pursue entrepreneurial activities that are unrelated to their current employment. In particular, career opportunities for young scientists at universities are limited, as there are few places in top academic positions and university employments are often limited in time. Thus, the following hypothesis can be formulated based on earlier findings:

(H3b): Job dissatisfaction has a moderating effect on the relationship between outcome expectations and entrepreneurial intention, such that when job dissatisfaction is high the relationship is stronger and when job dissatisfaction is low the relationship is weaker.

## Research methodology

### Data collection and sample

This contribution is based on cross-sectional data collected in a nationwide, online survey of academics at the seven public Swiss Universities of Applied Sciences. Since 1995, the UAS expanded its activities in research and development supported by the legal performance contract (Bundesgesetz über die Fachhochschulen, 1995). Intensive cooperation with non-institutional players in the context of practice- or business-oriented education and practice-oriented research remains central to the mission of UAS. In recent years, thus, the promotion of science-based start-ups has become an integral part of the service spectrum of universities in Switzerland. Accordingly, many universities and UASs have introduced measures to promote the commercialization of research. The pecuniary benefits of spin-offs, license rewards, and contract research have become an important source of income for Swiss universities. In addition, a large proportion of members of UASs have completed their academic education (doctorate, habilitation) at universities; we thus assume that our results are transferable to other research institutions and universities.

In Questback, an online survey tool (Unipark, 2013), participants could select from three languages (German, English, and French). Before conducting our study, we tested and optimized the questionnaire and procedures on an independent sample of academics from a large Swiss university. In January 2019, more than 8,900 academics from various disciplines were randomly invited via e-mail to participate in the survey. After eliminating incomplete responses, the final sample consists of 593 participants. The mean age of these respondents was 43.1 years (SD = 10.0, range: 25–69), 63.7% (n = 378) of these participants were male. Fifty percent (n = 289) of the participants belonged to STEM disciplines, including mathematics, computer science, natural science, and technology, while the others belonged to the social sciences and business administration. Academics who stated that they gained entrepreneurial experience were included as controls. An overview of the demographic characteristics of the sample is provided in Table 1. In January 2019, when the survey was conducted, all participants were working under contract at the university. The sample corresponds to the demographic distribution in terms of age, nationality, gender, and education of scientists at the Swiss UASs (Bundesamt für Statistik, 2019).

**Table 1:** Descriptive statistics of the sample (*n *= 593)

		Frequency	Percentage
Gender	Male	378	63.7
	Female	215	36.3
Discipline	Social Sciences	304	51.3
	Natural Sciences (STEM)	289	49.7
Academic status	Professor with leadership responsibility	180	30.4
	Professor without leadership responsibility	141	23.8
	Research assistant	272	45.9
Temp. work contract		222	37.4
Highest educational qualification	Master (UAS, university)	296	49.9
	Doctorate or post-doctoral degree	297	50.1
		Mean	SD
Age		43.1	10.0
Employment level		80.1	22.9

### Measures

#### Dependent variables

To test our hypothesis, this contribution relies on prior research in terms of capturing academics’ (a) general entrepreneurial intention and (b) spin-off intention (Goethner et al., 2012; Huyghe & Knockaert, 2015; Moog et al., 2015; Obschonka et al., 2012). Similar with previous entrepreneurial research studies, principal component analysis was used to investigate patterns by summarizing dominant gradients of variation in six response variables (described below). The first two principal components accounted for 80% of cumulative variance, showing a probable two factorial structure. Participants in the survey were offered a precise definition of spin-off activities in order to avoid possible confusion and inaccuracies in the measurement of spin-off intention. Spin-offs are based either on the intellectual property resulting from research or on skills and knowledge developed at a university. Intellectual property or skills are essential for the creation of the company (i.e., academic entrepreneurship).

##### Spin-off intention (SPIN)

To measure SPIN, three items were used as a seven-point Likert scale (1 = “very unlikely” to 7 = “very likely”). It was asked e.g. “You will engage in the establishment of a company based upon an idea, on knowledge or specific competencies or technology developed at the university,” based on Obschonka et al. (2015), with scale reliability measured by Cronbach's α = 0.85.

##### Entrepreneurial intention (EI)

Three items were used as a seven-point Likert scale (1 = “very unlikely” to 7 = “very likely”) to measure general EI, e.g., “You have the firm intention of becoming an entrepreneur one day,” based on Liñán and Chen (2009). Scale reliability, measured by Cronbach's α = 0.88, was above the generally accepted criterion of 0.70, indicating high reliability (Cortina, 1993).

#### Independent variables

##### Job satisfaction

Four items were averaged and used as a seven-point Likert scale (1 = “Absolutely incorrect” to 7 = “Absolutely correct”) to estimate job satisfaction of the respondents. E.g., “Overall, I am very pleased with the types of activities that I do in my job,” “Overall, I am very satisfied with my salary,” and “Overall, I am very pleased with my career opportunities,” adapted from Wanous et al. (1997) and Gagné et al. (2015). Scale reliability was acceptable, measured by Cronbach's α = 0.77.

##### Outcome expectations (OE)

Based on Miranda et al. (2018), we employed four items to measure OE as a seven-point Likert scale (1 = “Absolutely disagree” to 7 = “Absolutely agree”): (1) *Autonomy*: “Being an entrepreneur would entail a very high degree of Autonomy,” (2) *Profit*: “The financial return that I would get by becoming an entrepreneur would be high,” (3) *Self-realization:* “The personal satisfaction from being an entrepreneur would be very high,” and (4) *Quality of life:* “The quality of life that I would get from being an entrepreneur would be very high.” The four items were averaged based on scale reliability measured by Cronbach's α = 0.82.

##### Perceived behavioral control (PBC)

Three items were used as a seven-point Likert scale to measure PBC. The three items were (1) “I can control the creation process of a new company,” (2) “I know how to develop an entrepreneurial project,” and (3) “I know the necessary practical details to start a company” with scale reliability of Cronbach's α = 0.89 (Seven-point Likert scale; 1 = “Absolutely incorrect” to 7 = “Absolutely correct”).

#### Control variables

Multiple factors are recognized as influencing a scientist’s EI, including the following. *Gender* [women = 0, men = 1] was controlled for, as men are usually more entrepreneurially active (Zhao et al., 2005; Miranda et al., 2017; Abreu & Grinevich, 2017). Additionally, Goel et al. (2015) demonstrated a lower EI among female academics. *Nationality* [foreign = 0, Swiss citizen = 1] was controlled for, as individuals with foreign citizenship demonstrate higher entrepreneurial interests (Peroni et al., 2016). *Age* was controlled, as older academics may have gained more social capital (Goethner et al., 2012). Since there is a considerable body of research showing that social capital (in the sense of social networks) is associated with pecuniary resources and market knowledge, and thus a greater propensity to spin-off creation (Fernández-Pérez et al., 2014), *academic statu*s (professor [no = 0, yes = 1] (Huyghe & Knockaert, 2015; Goethner et al., 2012; Ucbasaran et al., 2008), *highest job qualificatio*n (master’s degree [no = 0, yes = 1], doctoral degree [no = 0, yes = 1] (Goethner et al., 2012; Huyghe & Knockaert, 2015), postdoctoral qualification [no = 0, yes = 1]), and *discipline* (social science and humanities = 0, STEM = 1) were accounted for (Abreu & Grinevich, 2014; Mosey & Wright, 2007; Krabel & Mueller, 2009). Additionally, the *level of employment* (in percent) and *temporary work contrac*t [no = 0, yes = 1] was controlled as a dummy variable, since limited work contract negatively predict job satisfaction (Waaijer et al., 2017). Following Huyghe and Knockaert (2015), we controlled for the *spin-off mission* of the universities, measured as a seven-point Likert-Scale. Bercovitz and Feldman (2008) emphasized that the individual behavior of academics is strongly affected by the social norms of the departments.

#### Data analysis

To test the hypotheses, the technique of structural equation modeling (SEM) using lavaan R package v. 0.6–5 (Rosseel, 2012) in R (R Core Team, 2013) was employed. This procedure uses fit indices to examine whether, and how well, the hypothesis-based model fits the data. Based on previous recommendations in social sciences (Kline, 2005), this study focused on the overall fit indices (Chi-Square Statistics, Root mean square of approximation RMSEA) and the incremental fit indices (Tucker Lewis Index = TLI, Comparative Fit Index = CFI). A non-significant $X$^2^ indicates a good fit, but using $X$^2^ alone as a fit statistic is problematic because it is influenced by the sample size and the extent of the correlations in the model. Generally, a CFI and a TLI of greater value than 0.90 indicate a reasonably good fit. In terms of the RMSEA, values ≤ 0.05 indicate a close approximation, and values between 0.05 and 0.08 indicate a reasonable approximation error (Kline, 2005).

#### Convergent, discriminant validity and common method variance

Before testing the hypotheses, confirmatory factor analysis was carried out to verify the distinctiveness of our measurements (discriminatory validity) and to estimate the effects of commonly measured variances. The criterion of Fornell and Larcker (1981) has commonly been used to assess the degree of shared variance between latent variables of the model, and it was used to test convergent validity. On the basis of a confirmatory factor analysis ($X$^2^[94.0] = 219.7 *p* < 0.001, RMSEA = 0.05, CFI = 0.97, TLI = 0.97), convergent validity can be investigated by calculating the Average Variance Extracted (AVE) using a cut-off point of 0.50 (Hair et al., 2017). The inspection of the AVE values (Table 2) for all factors suggests an acceptable convergent validity (AVE > 0.50, is considered as acceptable, AVE > 0.70 as very good).

**Table 2:** M and SD are used to represent mean and standard deviation, respectively. The values shown in bold are the square root of AVE. * indicates p < 0.05. ** indicates p < 0.01

Variable	*M*	*SD*	(1)	(2)	(3)	(4)	(5)
(1) Ent. Intention (EI)	2.39	1.46	**0.84**				
(2) Spin. Intention (SPIN)	2.58	1.57	0.70**	**0.90**			
(3) Perceived behavioral control	3.34	1.41	0.43**	0.35**	**0.83**		
(4) Job-Satisfaction	4.72	1.15	-0.09*	-0.02	-0.02	**0.74**	
(5) Outcome Expectation	3.65	1.24	0.55**	0.43**	0.37**	-0.07	**0.73**

Discriminant validity was evaluated in two ways. First, it was evaluated by comparing the constructs’ values of the squared root of the AVE ($\sqrt{\mathrm{AVE}}$) with the correlation of the other constructs (Fornell & Larcker, 1981) (see Table 2). A value of √AVE that is higher than the coefficient of the correlation between factors provides evidence of discriminant validity. As shown in Table 2, all factors met the criterion and demonstrated discriminant validity. Second, discriminant validity was evaluated by using a more recent technique, the heterotrait-monotrait ratio of the correlation (HTMT) (Henseler et al., 2015). HTMT is the average of the heterotrait-heteromethod correlation relative to the average of the monotrait-heteromethod correlation. If HTMT is below 0.90, a discriminatory validity between two reflective constructs can be assumed. Results show that the HTMT values between the respective constructs appeared to be below 0.90 (highest value of HTMT = 0.82 for the link between entrepreneurial and SPIN, lowest HTMT = 0.04 for perceived behavioral control and satisfaction). The results provide evidence for convergent and discriminant validity.

Common method variance (CMV) arises if a method bias influences all measures equally (Podsakoff et al., 2012) and can occur when respondents systematically distort their responses to surveys, e.g., according to social desirability. To examine the potential of CMV, all study variables were loaded onto one factor to examine the fit of the CFA model. If the one-factor CFA model fits the data, the common method variance is considered largely responsible for the relationship among the variables (Mossholder et al., 1998). The one-factor CFA model did not represent the data very well (χ^2^ (119) = 2563.8, *p* < 0.001, CFI = 0.55, RMSEA = 0.19), demonstrating that the study variables were not just different aspects of an underlying construct (CMV) (Fig. 1).

## Results

Table 3 presents the zero-order correlations with Bonferroni Correction between all variables used to investigate the prediction model for explanation of EI and SPIN. In line with the theoretical expectations, EI and SPIN are correlated with perceived behavioral control (r_Ent/PBC_ = 0.43, *p* < 0.001 and r_Spin/BC_ = 0.35, *p* < 0.001) (Table 3). EI and SPIN intention are positively correlated with outcome expectations (r_Ent/OE_ = 0.55, *p* < 0.001 and r_Spin/OE_ = 0.43, *p* < 0.001). No statistically significant correlation emerged between job satisfaction and the SPIN (H3a). Whereas, as expected in hypothesis (H3b), a negative correlation between the EI and satisfaction was observed (r_EI/Sat_ = − 0.9, *p* < 0.05). A high correlation between SPIN and EI (r_Spin/EI_ = 0.70, *p* < 0.001) is apparent in the data.

**Table 3:** Pearson correlation coefficients with pairwise-deletion and statistical significance based on Bonferroni Correction. * indicates p < 0.05. ** indicates p < 0.01

	(1)	(2)	(3)	(4)	(5)	(6)	(7)	(8)	(9)	(10)	(11)	(12)	(13)	(14)	(15)	(16)	(17)
(1) Ent. Intention	1																
(2) Spin. Intention	0.70**	1															
(3) Perceived behavioral control	0.43**	0.35**	1														
(4) Job-Satisfaction	-0.09*	-0.02	-0.02	1													
(5) Outcome Expectation	0.55**	0.43**	0.37**	-0.07	1												
(6) Uni. Spin-Mission	0.29**	0.38**	0.13**	0.08*	0.14**	1											
(7) STEM-Discipline	0.21**	0.28**	0.10*	-0.01	0.14**	0.25**	1										
(8) (Post-)Doctoral degree	-0.10*	-0.09*	-0.03	0.00	-0.07	-0.02	-0.08	1									
(9) Professor with leadership responsibility	-0.02	0.06	0.11**	0.09*	0.03	0.09*	0.03	0.08	1								
(10) Professor without leadership responsibility	-0.08	-0.09*	0.03	-0.05	-0.09*	-0.02	-0.16**	0.11**	-0.36**	1							
(11) Research assistant	0.09*	0.02	-0.13**	-0.05	0.04	-0.06	0.11*	-0.17**	-0.62**	-0.51**	1						
(12) Temp. work contract	0.15**	0.12**	-0.08	-0.04	0.07	0.07	0.05	-0.10*	-0.37**	-0.21**	0.52**	1					
(13) Employment level	0.02	0.10*	-0.04	0.02	0.01	0.05	0.26**	0.04	0.29**	-0.13**	-0.16**	-0.22**	1				
(14) Gender (men/woman)	-0.09*	-0.20**	-0.10*	-0.09*	-0.03	-0.11**	-0.26**	-0.05	-0.14**	-0.08	0.19**	0.10*	-0.25**	1			
(15) Age	-0.15**	-0.09*	0.13**	-0.04	-0.12**	-0.01	-0.11**	0.15**	0.41**	0.29**	-0.63**	-0.45**	0.06	-0.12**	1		
(16) Swiss citizens	-0.08	-0.07	0.02	0.01	-0.02	-0.04	-0.08*	-0.03	-0.02	0.01	0.01	-0.02	-0.10*	0.05	0.05	1	
(17) Ent. experience	0.36**	0.33**	0.31**	-0.12**	0.20**	0.08	0.08*	-0.04	0.10*	0.02	-0.11**	0.04	-0.07	-0.08	0.09*	0.00	1

### Testing the path model

The hypothetical model (H1) and (H2) was tested with perceived behavioral control and outcome expectations as predictors of EI and SPIN (all constructs were measured as latent variables in the model), including the control variables. Due to missing data (less than 3%), the full information maximum likelihood (FIML) estimation was used (Enders & Bandalos, 2001). The model fit was acceptable ($X$ ^2^ [168] = 407.7, *p* < 0.001, RMSEA = 0.05, CFI = 0.95, TLI = 0.94), indicating that the measurement of the latent variables was sound.

The model explained 55% of the variance of EI and 44% of the variance of SPIN. Perceived behavioral control had a significant effect of $\beta = 0.21 \left(p<0.001\right)$ on EI and a significant effect of $\beta = 0.14 \left(p<0.001\right)$ on SPIN, indicating support for (H1a) and (H1b). Also, corresponding with hypotheses (H2a) and (H2b), the results show that outcome expectations have a direct effect on both the EI $(\beta = 0.52, p<0.001)$ and SPIN $(\beta = 0.36, p<0.001)$. Among the control variables, gender, entrepreneurial experience, and university spin-off mission positively affected both entrepreneurial intention and spin-off intention. STEM-Discipline showed a positive effect on SPIN. The effects of the control variables on EI and SPIN are shown in Table 4*.*

**Table 4:** Structural model path coefficients, R^2^, and fit statistics for the models. EI – Entrepreneurial Intention, SPIN – Spin-off intention

Path			Research Model *Overall (n* = *593)*		Low Job-Satisfaction *(n*_*Low*_ = *332)*		High Job-Satisfaction *(n*_*High*_ = *261)*	
			St. path coefficient	*p*	St. path coefficient	*p*	St. path coefficient	*p*
Outcome Expectation	--- >	EI	0.52	< 0.001	0.55	< 0.001	0.47	< 0.001
Perceived behavioral control	--- >	EI	0.21	< 0.001	0.23	< 0.001	0.17	< 0.001
Job-Satisfaction	--- >	EI	- 0.02	0.59				
Uni. Spin-Mission	--- >	EI	0.20	< 0.001	0.16	< 0.001	0.28	< 0.001
Gender women	--- >	EI	- 0.04	0.22	- 0.08	0.09	0.01	0.93
Age	--- >	EI	- 0.09	0.05	- 0.09	0.14	- 0.09	0.18
Professor	--- >	EI	0.04	0.29	0.06	0.24	0.03	0.61
Entrep. experience	--- >	EI	0.23	< 0.001	0.27	< 0.001	0.20	< 0.001
Employment level	--- >	EI	0.04	0.33	0.02	< 0.05	- 0.06	0.31
Nationality	--- >	EI	- 0.03	0.33	0.02	0.68	0.09	0.08
Temp. work contract	--- >	EI	0.08	0.05	0.08	0.16	0.11	0.09
(Post-)Doctoral degree	--- >	EI	0.10	0.05	0.04	0.78	0.16	0.30
STEM-Discipline	--- >	EI	0.40	0.28	0.07	0.17	0.03	0.60
*R*^*2*^		EI	0.55		0.62		0.54	
Outcome expectation	--- >	SPIN	0.36	< 0.001	0.37	< 0.001	0.36	< 0.001
Perceived behavioral control	--- >	SPIN	0.14	< 0.001	0.14	< 0.01	0.12	0.08
Job-Satisfaction	--- >	SPIN	- 0.05	0.24				
Uni. Spin-Mission	--- >	SPIN	0.30	< 0.001	0.30	< 0.001	0.34	< 0.001
Gender women	--- >	SPIN	- 0.10	< 0.05	- 0.15	< 0.05	- 0.05	0.39
Age	--- >	SPIN	- 0.08	0.09	- 0.18	< 0.05	0.03	0.70
Professor	--- >	SPIN	0.04	0.51	- 0.03	0.61	0.02	0.77
Entrep. experience	--- >	SPIN	0.24	< 0.001	0.31	< 0.001	0.13	< 0.05
Employment level	--- >	SPIN	0.04	0.33	0.11	< 0.05	0.01	0.83
Nationality	--- >	SPIN	- 0.03	0.47	- 0.02	0.65	- 0.02	0.65
Temp. work contract	--- >	SPIN	0.10	< 0.05	0.08	0.15	0.11	0.09
(Post-)Doctoral degree	--- >	SPIN	0.10	0.35	0.08	0.35	0.13	0.42
STEM-Discipline	--- >	SPIN	0.10	< 0.05	0.06	0.23	0.13	< 0.05
*R*^*2*^		SPIN	0.44		0.48		0.42	
*Model Fit*								
X^2^	=	407.7	df = 186	< 0.001	608.9	df = 336	< 0.001	
RMSEA	=	0.046			0.053			
CFI	=	0.941			0.942			
TLI	=	0.923			0.928			

### Testing the moderation

In order to test the moderating effect of job satisfaction, a SEM multi-group analysis in lavaan R package v. 0.6–5 (Rosseel, 2012) was conducted. By applying a median split of the aggregated items of job satisfaction (Mean = 5.1, SD = 1.12, Median = 5.2), two groups were created (high job satisfaction; n = 261 and low job satisfaction; n = 332. For this analysis, the items of the outcome expectations scale were aggregated. This procedure was applied previously in other entrepreneurship studies (e.g., Obschonka et al., 2012).

Subsequently, a number of mean difference tests of the manifest variables of each scale (e.g., mean value of the EI) were performed. The two groups did not differ in terms of the dependent variables *EI* (t[550.4] = 0.96, *p* = 0.35), *SPIN* (t[549.3] = 0.37, *p* = 0.71), *gender* (${X}^{2}\left[1\right]= 0.43, p= 0.51)$, and *discipline* (${X}^{2}\left[1\right]= 0.30 p= 0.58)$. The dissatisfied academics did not have statistically significant higher values for outcome expectations (Mean = 4.05, SD = 1.17) than the highly satisfied academics (Mean = 3.9, SD = 1.28; t[518.1] = 1.50, *p* = 0.13). According to the multi-group model outcome expectations showed a significant effect of β_LowSatisfation_ = 0.55 (*p* < 0.001) on EI among academics with low job satisfaction and an effect of β_HighSatisfaction_ = 0.47 (*p* < 0.001) on EI among academics with high job satisfaction, indicating a negative moderating effect of job satisfaction (Table 4). The effects of outcome expectations on SPIN did not essentially differ within the low job satisfaction group (β_LowSatisfation_ = 0.37, *p* < 0.001) compared to the group with high job satisfaction (β_HighSatisfaction_ = 0.36, *p* < 0.001).

The group of low job satisfaction demonstrated a lower correlation between EI and SPIN (r_LowSatisfaction_ = 0.65, *p* < 0.001) compared to the group with high job satisfaction (r _HighSatisfaction_ = 0.76, *p* < 0.001) suggesting that the perception of differences between the two constructs increases with higher levels of job dissatisfaction.

In terms of variance elucidation, dependent and control variables explained more variance in EI (R^2^_LowSatisfaction_ = 0.60, R^2^_HighSatisfaction_ = 0.54) compared to the explained variance in SPIN (R^2^_HighSatisfaction_ = 0.47, R^2^_LowSatisfaction_ = 0.42).

**Fig. 2:**
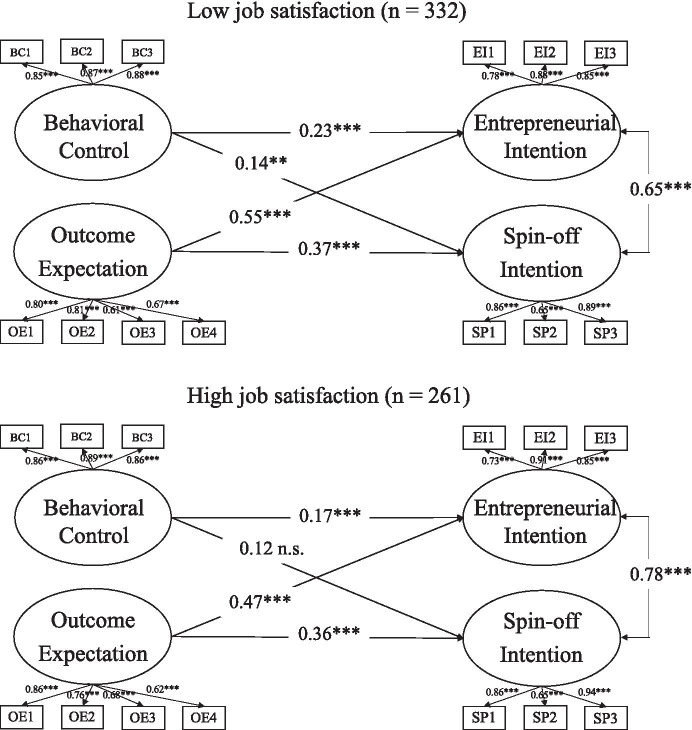
Multi-group analysis (Moderator: Job Satisfaction), Behavioral Control. Note: Standardized coefficients are given. All effects are controlled for gender, field, nationality, venture already founded, academic status, qualifications and contract of employment. *p < 0.05, **p < 0.01, ***p < 0.001

Next, differences in job satisfaction between the two groups were evaluated. A Chi-square difference test revealed that the unconstrained and constrained (factor-loadings, measurement intercepts) did not differ in their fit ($\Delta {X}^{2} \left[16\right]=22.0, p=0.15)$, indicating measurement invariance across both groups. The next step was to test the unconstrained model against models, where one of the paths was always set equal across both groups (see Fig. 2). A significant moderating effect in the case of the link between outcome expectations and EI was revealed, but not in the case of outcome expectations and SPIN (as indicated by the significant $\Delta {X}^{2}$). While evidence in support of hypothesis (H3a) was weak, the moderation analysis indicated a negative moderation effect of job satisfaction on the relationship between outcome expectations and EI in support of hypothesis (H3b) (Table 5).

**Table 5:** Fit indices and ${\mathrm{X}}^{2}$ difference test for moderation effect of job satisfaction. Note *p < 0.05, **p < 0.01, ***p < 0.001

Models – Moderation	$X$^2^	$df$	CFI	RMSEA	$\Delta X$^2^	$\Delta df$
Unconstrained model I	609***	336	0.94	0.053		
Perceived behavioral control ➔ EI set equal across groups	610***	337	0.94	0.052	1.31	1
Perceived behavioral control ➔ SPIN set equal across groups	609***	337	0.94	0.053	0.22	1
Outcome Expectation ➔ EI set equal across groups	615***	337	0.94	0.053	5.5*	1
	610***	337	0.94	0.053	0.51	1
Outcome Expectation ➔ SPIN set equal across groups						

### Exploratory analysis of outcome expectation on the spin-off and entrepreneurial intention

Associations between the outcome expectations and entrepreneurial and spin-off intentions were examined (see Fig. 2). The items of outcome expectations for autonomy, profit, satisfaction, and quality of life were individually included as independent variables in the model (additionally, the control variables and perceived behavioral control), resulting in strong model fit ($X$ ^2^ [157] = 365 *p* < 0.001, RMSEA = 0.05, CFI = 0.96, TLI = 0.94). Regarding EI, the empirical model indicated a direct effect of outcome expectation self-realization $(\beta = 0.31,p<0.001)$ and outcome expectation quality of life $(\beta = 0.27,\mathrm{ p}<0.001)$ on entrepreneurial intention. By contrast, no effect of outcome expectations autonomy $(\beta = - 0.06, p=0.11)$ and outcome expectations financial profit $(\beta = 0.04, p=0.92)$ was evident for EI. Regarding SPIN, besides outcome expectations self-realization $(\beta = 0.16 , p<0.01)$ and outcome expectations quality of life $(\beta = 0.18, p<0.001)$, no effect of outcome expectations profit (*β* = 0.07, $p$ = 0.13) or outcome expectations autonomy (*β* = − 0.04, $p$ = 0.34) was statistically significant. These results suggest that both entrepreneurial and spin-off intentions are driven by an expectation of improvement in the quality of life and self-realization.

## Discussion

Our study examined the motivation and subjective perception of the environment in which an academic researcher considers their potential career as an entrepreneur. We investigated academics from Swiss Universities of Applied Sciences, obtained from an online survey conducted in Spring 2019. The study’s results enhance our understanding of the relationship between job-satisfaction and entrepreneurial career decisions among researchers, making an important distinction between entrepreneurial intention and spin-off intention. Academic spin-offs (based on intellectual property or knowledge and skills) are considered to be a specific form of academic entrepreneurship. They are an essential part of the commercial knowledge transfer, a vital task of entrepreneurial universities (Etzkowitz, 2017; Meek and Wood, 2016) and thus a considerable extension of an academic career. By providing insight into entrepreneurial intention we gained a greater understanding of general entrepreneurial activities that extend beyond the academic context as well as activities of knowledge transfer.

Little is currently known about the motivational factor driving entrepreneurial career decisions among researchers. This is particularly true when it comes to the differences in academic entrepreneurship (i.e., spin-offs) and other forms of entrepreneurial activities among academic researchers. We revealed that the relationships between outcome expectations and entrepreneurial decisions were variable and often context dependent. Generally, academic researchers in our sample showed a high level of job satisfaction. A multi-group analysis revealed no statistically significant moderating effects of job satisfaction on the relationship between outcome expectation and spin-off intention. However, a moderating effect of job dissatisfaction between outcome expectation and entrepreneurial intention was evident, leading to two possible explanations. First, it is possible that participants perceived entrepreneurial careers and spin-off careers as distinct alternatives. Second, as hypothesized, different psychosocial micro-processes may be involved when studying academic entrepreneurship in the form of spin-offs compared to general entrepreneurial decisions among academic researchers.

The results from this study provide new evidence for the importance of accurate operationalization of entrepreneurial action and the need to clearly distinguish entrepreneurial intentions, e.g., for spin-offs, from other forms of entrepreneurial action. Spin-offs are considered a specific case of entrepreneurship, but entrepreneurial intention may also include extramural forms of entrepreneurship and thus modes of entrepreneurship which are not related to knowledge transfer. Our results add to the literature in that entrepreneurial-minded researchers are more likely to engage in entrepreneurial activities, rather than spin-off activities if they are dissatisfied with their current employment. This implies that previous findings from the entrepreneurship literature, which demonstrated that job-dissatisfaction increases the probability of the transition to self-employment (Chang & Edwards, 2015; Guerra & Patuelli, 2016; van Dick et al., 2004; Werner et al., 2014), may also be equally applicable to academic researchers.

Recently published research has postulated a relationship between job satisfaction, organizational commitment, and entrepreneurial activities (Singh & Onahring, 2019). Our results do not support the assumed effect that entrepreneurial researchers with high job satisfaction develop a more substantial interest in spin-off activities. It could be argued that scientists who are satisfied with their current position may seek to maintain the status quo and not pursue additional spin-off activities. Prior qualitative research has shown that academics are developing a second identity as entrepreneurs within the entrepreneurial university, alongside their traditional academic identity (Boffo et al., 2019). The two forms of identity may converge when the traditional scientific identity also benefits from the entrepreneurial role's successes. However, a key criterion for scientific identities to become more entrepreneurial would be for universities to broaden their goals in terms of spin-off activities beforehand. In the current study, only thirty-six percent of respondents stated that their universities would significantly or partially support spin-offs activities of researchers. We, therefore, assume that spin-off activities do not yet have a high priority at all higher education institutions, and that success in spin-off activities may not yet strengthened academic careers.

We also argue that perceived feasibility in the form of perceived behavioral control does matter when deciding to move into entrepreneurship. Ajzen (2002) considered perceived feasibility in form of the concept of self-efficacy as part of a superordinate construct of perceived behavioral control. While mostly measured as a one-dimensional construct, self-efficacy was shown to be a significant predictor of entrepreneurial intention in prior academic entrepreneurship research (Díaz-García & Jiménez-Moreno, 2010; Guerrero et al., 2008; Huyghe & Knockaert, 2015). Per our results, entrepreneurial intention, as well as spin-off intention, were positively influenced by perceived behavioral control. Our results indicate that participants with a higher level of perceived behavioral control showed a greater likelihood to develop entrepreneurial as well as spin-off intentions when controlling for prior entrepreneurial experience and other personal control variables (e.g., age, discipline, and gender, entrepreneurial mission of the university). This finding is consistent with prior entrepreneurship literature (Brettel et al., 2013; Díaz-García & Jiménez-Moreno, 2010; Krabel & Mueller, 2009; Moog et al., 2015; Obschonka et al., 2010, 2012, 2015). Our study therefore supports perceived behavioral control as an interesting construct to study beliefs of dealing with ease or difficulties in entrepreneurial task performance.

However, our study adds to the literature with evidence that the relevance of perceived behavioral control for spin-off intention is weaker than for other entrepreneurial activities. Our analysis revealed that perceived behavioral control has a greater, albeit slight, influence on predicting entrepreneurial intentions when compared to spin-off intentions. Spin-offs are more likely to manifest in research teams as it reduces individual pressures to manage every step to become a successful founder. Also, universities, technology transfer offices, science parks, and incubators offer opportunities, such as coaching and training to acquire skills needed to create spin-off activities, suggesting less responsibility for the individual. Previous research has shown that these opportunities have a positive effect on perceived behavioral control (Miranda et al., 2017). One likely explanation for this outcome is that individuals may not need to rely exclusively on their skills when selecting a spin-off career, because spin-offs are mostly founded by teams rather than individuals. Therefore, feasibility may play a less crucial role in spin-off decisions than in other forms of self-employment. We urge future research to investigate the role of individuals' perceived behavioral control in early entrepreneurial teams.

With respect to personal motivation, we tested the hypothesis articulated in SCCT that outcome expectation would be a predictor for entrepreneurial intention. We noted a positive effect of outcome expectation on both entrepreneurial intention and spin-off-intention, suggesting that higher outcome expectations encourage a transition into entrepreneurship. However, the effect of outcome expectation was more important for predicting entrepreneurial intention than for spin-off intention. Our analysis also revealed that expected profit and autonomy were not significant motivations for spin-off or entrepreneurial intentions. Furthermore, self-realization and expected improvements in quality of life explained entrepreneurial intention more reliably when compared to spin-off intention. When considering other outcome expectations, we found that reputation and extrinsic rewards were stronger predictors of spin-off decisions than self-realization and quality of life. This finding is consistent with postulates from SCCT and the literature that suggests that motivations in the form of specific outcome expectations explain entrepreneurial career decisions (Antonioli et al., 2016; Goethner et al., 2012; Guerrero & Urbano, 2014; Lam, 2015; Miranda et al., 2017; Morales-Gualdrón et al., 2009). However, our results show that entrepreneurial career decisions are not necessarily linked to expected pecuniary gains as a primary goal. Other scholars referred to additional pecuniary advantages such as compensation for their time and efforts spent on entrepreneurial activities driving intentions rather than pure motivation (Hossinger et al., 2020; Morales-Gualdrón et al., 2009).

Our study provides evidence that the probability of a spin-off intention is positively influenced by previous entrepreneurial experience, gender (e.g., women show a lower level of spin-off intention), fixed-term employment contracts, employment in the STEM disciplines, and a perceived spin-off mission by the university. By contrast, age, academic status, level of employment (in percent), nationality, and highest degree obtained did not significantly account for spin-off intention. These findings support the results of prior research where entrepreneurial intention has been found to be positively influenced by prior entrepreneurial experience and an explicit spin-off mission of the university, suggesting that the promotion of an entrepreneurial mission within universities contributes significantly to spin-offs and to other entrepreneurial activities (e.g., Huyghe & Knockaert, 2015; Foo et al., 2016).

### Limitations and further research

Analysis of academic entrepreneurship and job satisfaction is prone to several well recognized limitations, which ultimately inform possible avenues for future research. One consideration is our survey data, despite representing a large sample size, refers specifically to the context of Swiss UAS in 2019. In Switzerland, salaries are comparatively high, and researchers have opportunities to switch into industry, which must be considered when interpreting results regarding outcome expectations and entrepreneurial intention. The results may also be viewed as lacking generality because UASs demonstrate atypically strong ties to industry compared to other countries, leading to greater opportunities for entrepreneurial activities on the margins of academic employment. Future studies should focus on other countries with lower opportunity costs for entrepreneurial activities and higher unemployment rates to study the relationship between job-satisfaction and entrepreneurial activities among academics. In this study, a distinction was made between entrepreneurial intentions and spin-off intentions by assuming that spin-off intentions were a specific case of entrepreneurial intentions. Additional research distinguishing between spin-off intentions and extramural forms of entrepreneurship is warranted and could yield more contrasting outcomes.

This study was designed to generate cross-sectional data, this longitudinal data to assess the impact of possible interactions between organizational conditions and academic entrepreneurial behavior may be insightful. In particular, longitudinal studies could elicit the extent to which spin-offs and start-ups arise from long-term job dissatisfaction. Additionally, future studies are urged to follow a multi-level approach and test the extent to which different academic positions and team-related factors influence entrepreneurial behavior. A methodological limitation of this study was the lack of validated measurement scales. In particular, a re-validation of the job satisfaction scales within academia is required for future studies. Additionally, a mixed-methods approach could be used to investigate the extent to which spin-off careers are seen as alternative career paths and whether they could be developed by training and a stronger presence of role models.

## Conclusion

This study contributes new knowledge to the existing literature on the determinants of entrepreneurial activity among academics in three specific areas. First, this study demonstrates that job-dissatisfaction fosters the re-evaluation of outcome expectations to define entrepreneurial career goals. Our data did not confirm a positive moderating effect of job-satisfaction on the relationship between outcome expectations and spin-off intention. Taken together, our results support the role of job satisfaction as an interesting variable in academic entrepreneurship research. Second, the results emphasize that entrepreneurial activities are associated with specific expectations, including improvements in self-realization and quality of life. Individuals make critical choices between employment and self-employment to maximize the benefits of career choice when considering expected outcomes (Douglas & Shepherd, 2000). Third, this paper represents an empirical application of the framework of SCCT, as recommended by Liguori et al. (2018) and Tran and Korflesch (2016), to investigate academic entrepreneurship. This contribution also addresses the recommendations of Singh and Onahring (2019) to examine the relationship between job satisfaction and entrepreneurial intention. Our analysis emphasized that several motives are driving the entrepreneurial goals among academics that warrant further research. Overall, this study underlines the importance of individual outcome expectations and perceived behavioral control, which merits greater attention by practitioners and knowledge-transfer agencies. In conclusion, pecuniary satisfaction is not the primary motive for a scientist to become entrepreneurial. Instead, non-pecuniary satisfaction such as personal fulfillment of one's ideas in combination with job-dissatisfaction proved to be a more compelling motivator.

## Data Availability

https://doi.org/10.17605/OSF.IO/ZB4WV; Link: https://osf.io/zb4wv/

## Footnotes

1 The Global Entrepreneurship Monitor defines entrepreneurship as “*any attempt at new business or new. venture creation, such as self-employment, a new business organization, or the expansion of an existing. business, by an individual, a team of individuals, or an established business*” (Bosma et al., 2012, p. 9).
